# Investigating mobile element variations by statistical genetics

**DOI:** 10.1038/s41439-024-00280-1

**Published:** 2024-05-30

**Authors:** Shohei Kojima

**Affiliations:** Genome Immunobiology RIKEN Hakubi Research Team, RIKEN Center for Integrative Medical Sciences, Yokohama, 230-0045 Japan

**Keywords:** Structural variation, Genome-wide association studies

## Abstract

The integration of structural variations (SVs) in statistical genetics provides an opportunity to understand the genetic factors influencing complex human traits and disease. Recent advances in long-read technology and variant calling methods for short reads have improved the accurate discovery and genotyping of SVs, enabling their use in expression quantitative trait loci (eQTL) analysis and genome-wide association studies (GWAS). Mobile elements are DNA sequences that insert themselves into various genome locations. Insertional polymorphisms of mobile elements between humans, called mobile element variations (MEVs), contribute to approximately 25% of human SVs. We recently developed a variant caller that can accurately identify and genotype MEVs from biobank-scale short-read whole-genome sequencing (WGS) datasets and integrate them into statistical genetics. The use of MEVs in eQTL analysis and GWAS has a minimal impact on the discovery of genome loci associated with gene expression and disease; most disease-associated haplotypes can be identified by single nucleotide variations (SNVs). On the other hand, it helps make hypotheses about causal variants or effector variants. Focusing on MEVs, we identified multiple MEVs that contribute to differential gene expression and one of them is a potential cause of skin disease, emphasizing the importance of the integration of MEVs in medical genetics. Here, I will provide an overview of MEVs, MEV calling from WGS, and the integration of MEVs in statistical genetics. Finally, I will discuss the unanswered questions about MEVs, such as rare variants.

## Genetic insights: mobile element variations influence human complex traits

This research investigates how mobile elements contribute to human genetic diversity and disease. Researchers conducted a review using various sequencing technologies to analyze MEs in different human populations, focusing on three main families: *Alu*, LINE-1, and SVA. They studied these elements in over 2,500 individuals worldwide, finding that MEs significantly contribute to genetic variation and are linked to certain diseases. Using the short-read sequencing method, they identified thousands of ME variations, some linked to genetic disorders. These methods help uncover how MEs affect genome structure and function. The results show that MEs account for a large part of human structural variations, influencing gene expression and contributing to populations’ genetic makeup. Future studies may clarify how MEs contribute to genetic diversity and disease, leading to new diagnostic and therapeutic strategies. This summary was initially drafted using artificial intelligence, then revised and fact-checked by the author.

## Mobile element variations in humans

Changes in genomes are triggered by mutagens. Mobile elements, sequences that change location within genomes, act as mutagens modifying the genomes, contributing to human genome variation^1^. *Alu*, LINE-1, and SVA are the three mobile element families that include recently active human mobile element lineages. More specifically, the *Alu* Y, L1HS, and SVA-E and F subfamilies contain human-specific lineages that are active in modern humans^2–8^. The lengths of *Alu* Y and L1HS are ~0.3 and 6.0 kb, respectively^2,4^, while SVA copies often show variable sizes between 0.7 and 4 kb^8^. These families are retrotransposons that mobilize and increase their copy number via a so-called “copy-and-paste” mechanism. *Alu* is transcribed by RNA polymerase III^9^, while LINE-1 and SVA are transcribed by RNA polymerase II^8,10,11^. Their RNA can be reverse transcribed into cDNA and inserted elsewhere within the genome by endonuclease and reverse transcriptase activities of a protein encoded by LINE-1^7,12–14^. Mobilization can occur in both germline and somatic cells; mobile elements are mutagens that have been inherited from ancestors. Multiple mechanisms suppress mobilization and protect the genome from insertions, such as epigenetic modifications and piRNA (reviewed in^15^); however, new mobilizations can still be observed.

Evolutionarily recent mobilizations have contributed to human genome diversity^16–19^. Some copies of human mobile elements show insertional polymorphisms between humans^5,8,20^. For example, 146,106 *Alu* Y, 1,204 L1HS, 328 SVA_E, and 393 SVA_F copies were found in the reference human genome GRCh38 (using RepeatMasker version 4.1.1^21^ and Dfam 3.2^22^). Of these, 2027, 225, 27, and 47 copies, respectively, were found to be absent in at least one individual of 2504 global humans recruited and sequenced at high depth (×30) by the 1000 Genomes Project^23^. In addition to their absence, non-reference insertions of 32,024 *Alu* Y, 6,521 L1HS, and 1,555 SVA were also found in at least one of the 2504 individuals. In this review, we refer to these insertionally polymorphic copies as mobile element variations (MEVs). Most MEVs likely arose after chimpanzee-human divergence due to their human specificity, though some may predate this. An analysis of 39 chimpanzee genomes revealed 15 MEVs shared with humans, 5 of which were found in the HLA locus, which is known for harboring haplotypes maintained by balancing selection^23^. A long-read resolved catalog of structural variants revealed that MEVs contribute to 25% of human SVs^19^. The principal components of MEVs in humans worldwide are similar to those of SNVs and capture the continental structure of human populations, suggesting that mobile elements played a role in diversifying the human genome during migration out of Africa^23^. Some mobile element lineages are still active even now. Our previous study identified 17,608 rare insertions (with an allele frequency (AF) < 1%) and 22,006 singletons from the 1000 Genomes Project. A substantial percentage of those MEVs (64%) were population specific, suggesting their recent occurrence. Genome analyses of pedigrees estimate a de novo mobilization rate of roughly one event per 20 births^24^. In addition to the three mobile element families, some copies of human endogenous retrovirus-K (HERV-K) exhibit insertional polymorphisms^25–27^. Although HERV-K was found to be active during human evolution, there was no clear evidence of its de novo mobilization in a family-based study^24^. The number of insertionally polymorphic HERV-K copies was orders of magnitude less than that of the other three mobile element families (i.e., *Alu*, LINE-1, and SVA)^25^.

Some mobile element families are involved in human traits and disease. Similar to single nucleotide mutations disrupting coding sequences, such as missense mutations introducing stop codons or frameshifts, mobile element mobilization can also result in loss-of-function (LoF) mutations. An SVA insertion in *SRCAP* causes the depletion and exon skipping of its transcripts, leading to neurodevelopmental disorders^28^. A LINE-1 insertion in *CC2D2A* generates the LoF allele, which leads to Joubert syndrome and ciliopathy^29^. Mobile element insertion in noncoding sequences, such as UTRs and introns, can also have a significant impact on disease through aberrant splicing and destabilization of transcripts. For example, an SVA insertion in the 3’-UTR of a gene encoding the fukutin protein is the most frequent cause of Fukuyama-type congenital muscular dystrophy^30^. An SVA insertion was found in an intron of *VHL* in a Von Hippel–Lindau patient, although its causal role requires further investigation^31^. Sequence variations in MEVs also influence disease. An SVA insertion in an intron of *TAF1* downregulates its expression and results in X-linked dystonia-parkinsonism^32,33^. The length of (CCCTCT)n repeats in the SVA inversely correlates with the age at onset^34^. These examples, mostly rare variants causing rare diseases, emphasize that investigations of MEVs can help identify the cause of various genetic diseases^35^. In addition to rare MEVs, common MEVs (e.g., AF > 1%) are also linked to diseases and traits. PCR-based genotyping of *Alu* variants revealed 33 elements tagged with trait-associated SNVs^36^. A common *Alu* variant (AF 69%) near an antiviral gene, *TRIM25*, is associated with chromatin states during virus infection^37^. An *Alu* variant (AF 1%) is linked to the expression of the oncogene *PTK2*, potentially through enhancer-promoter chromatin looping^38^.

## Identification and genotyping of mobile element variations

The accurate discovery and genotyping of variants is important for genetics. Particularly in statistical genetics, accurate genotyping is key for successful haplotype phasing, genotype imputation, and detection of genotype‒phenotype associations for SVs, including MEVs. While long-read sequencing technologies, such as PacBio and Oxford Nanopore Technologies, are promising approaches for accurately identifying SVs, Illumina and other short-read technologies remain the current standard for biobank-scale genome sequencing cohorts. Therefore, optimizing methodologies for both long- and short-read applications is beneficial for the integration of SVs in statistical genetics. Although there is currently no well-established practice for finding MEVs accurately at the population scale, several studies have successfully integrated MEVs into statistical genetics analyses^19,23,36,39,40^.

Short-read sequencing has been a standard for population-scale genome sequencing, and most of the WGS datasets in existing biobanks are short reads. In the case of short reads, it is common to first find reads that support breakpoints and then determine whether a given breakpoint is an MEV. There are several tools for making use of such genome datasets for MEV research. MELT is a software package that discovers and genotypes nonreference MEVs^41^. MELT was developed as a part of the 1000 Genomes Project and has been widely used to discover MEVs. The x-Transposable element analyzer (xTea) stands out for its flexibility, working with short and long reads, WGS and exome data^42^. Notably, it has been used extensively for the discovery and characterization of SVA variations^43^. TypeTE focuses on improving the genotyping of MEVs discovered by other tools^44^. We recently developed another tool, named MEGAnE, which is applicable to short-read WGS. MEGAnE has improved genotyping accuracy and can be used for statistical genetics. The VCF file output from MEGAnE can be merged with VCF files containing SNVs and indels and can be used for haplotype phasing and genotype imputation. This allows us to integrate MEVs into eQTL analysis and GWAS. In addition to those tools, many tools, such as Retroseq^45^, Mobster^46^, STEAK^47^, ERVcaller^48^, and TranSurVeyor^49^, are also available. However, limitations compared to long reads exist. MEV calling highly depends on mappability, and MEVs in complicated genome regions are hard to call from short reads. Additionally, fully determining the sequences of non-reference mobile element copies is often challenging with short reads alone.

Long-read sequencing is a promising technology for identifying SVs at the population scale. In 2021, the genomes of 35 global humans were analyzed with multiple sequencing technologies, including long reads. Using Phased Assembly Variant Callers (PAV), the authors first assembled haplotypes and called SVs, including MEVs. Subsequent annotation of MEVs in VCF output files from PAV was performed with a tool called MEIGA-PAV^19^. Tools specific for MEV discovery are also available for long reads. Realignment-based Mobile Element insertion detection Tool for Long read is a bioinformatic tool for discovering MEVs from PacBio and Oxford Nanopore (ONT) long reads^50^. xTea can be applied to both short and long reads^43^. The use of long-read sequencing in biobanks is expanding. Notably, ONT was applied for more than 3000 Icelanders, and the All of Us Research Program plans to generate WGS data using PacBio long reads.

Long reads have several advantages over short reads. First, long reads can detect variations inside mobile elements. Mobile element copies themselves can carry internal variations. For example, deletion of the internal region of full-length HERV results in the generation of a solo-LTR construct, and some SVA copies have SVs in variable nucleotide tandem repeats (VNTRs)^43,51,52^. Understanding such internal variation is an advantage in functional genomics, as it provides a mechanistic explanation of how MEVs are involved in disease and complex traits. However, it is difficult to call SNVs, indels, and SVs nested in mobile element copies from short reads because they often fail to be accurately mapped to recently active mobile elements due to multiple mapping problems. In contrast, such variations can be detected in long-read haplotype assemblies^19^. Furthermore, long reads can detect chromosomal rearrangements via mobile elements and noncanonical insertions. Mobilization occasionally triggers chromosomal rearrangements, for example, in cancers^53,54^. It is also known that mobile elements occasionally form complex sequence architectures, such as 5’- and 3’-transduction and 5’ inverted poly-A tails^8,55–57^. Such information can reveal the locations of active copies and the mobilization history, which are important for understanding the biology and pathogenesis of mobile elements^19,43,53^.

In addition to de novo variant calling, genotyping known SVs, including MEVs, from short-read data is also possible. PanGenie is a tool that takes short-read WGS data as the input and genotypes SVs that are found from long-read data^58^. If the purpose of an analysis is to evaluate common variants and a catalog of common SVs is available from a matched population, genotyping of known variants is an alternative approach.

Although some studies use deeply-sequenced genomes, for example, NA12878 and an Ashkenazim trio in the Genome in a Bottle Consortium^59^, as a ground truth to benchmark MEV callers, a side-by-side comparison at a population scale (e.g., more than 100 genomes per population) is lacking, making it difficult to find the best MEV caller and option for statistical genetics. In practice, accuracy is influenced by multiple WGS factors, such as the genome build, sequencing depth, read length, insert size, and alignment software. Therefore, experimentation with multiple tools and settings is crucial for optimizing genotyping methodologies for each specific cohort.

## Statistical genetics with mobile element variations

Statistical genetics can identify genome loci and variants associated with complex traits and disease. While SVs contribute to complex traits and diseases, current GWAS primarily focus on SNVs and short indels, overlooking SVs. Recent advancements in long-read sequencing have enabled accurate identification and genotyping of SVs at the population scale^19^. Additionally, progress in MEV calling from short reads also allows us to study MEVs within existing biobank WGS^23^. These technical advances have allowed us to integrate SVs, including MEVs, in the framework of statistical genetics, shedding light on medically relevant SVs previously missed by GWAS.

MEVs prepared in VCF format can be merged with SNVs. Using a merged VCF file, we can perform haplotype phasing, genotype imputation, and association testing. Even after quality control measures such as excluding variants violating the Hardy‒Weinberg equilibrium, we expect differing distributions of genotyping accuracy for SNVs and MEVs, as they are usually called by different algorithms. In the case of variant calling from short reads, SNVs tend to have a higher genotyping accuracy than SVs^60^. Consequently, this disparity must be considered when comparing SNVs and MEVs, as it can introduce confounding factors such as skewing statistical fine-mapping^61^. Therefore, careful interpretation is required when comparing statistics (e.g., posterior probabilities) between SNVs and MEVs.

The integration of MEVs in eQTL analysis allows for the evaluation of associations with gene expression. eQTL analysis of MEVs has been reported for iPSCs^39^, lymphoblastoid cell lines^19,39,62,63^, whole blood from Parkinson’s disease patients^64^, macrophages^37^, and 49 tissues collected by the GTEx consortium^23,40,65,66^. Recently, we discovered 1073 eQTLs with MEVs from GTEx data. Since there are fewer MEVs than SNVs, few MEVs are detected as sentinel (*n* = 384), and the majority of MEVs detected in eQTLs are tagged with non-MEV lead variants (*n* = 689, R^2^ > 0.95). While most of the causality of MEVs has not been evaluated by wet experiments, some MEVs have been validated to be effectors. For example, *Alu* variants in the 3’-UTR of *HSD17B12*, *ADIPOQ*, and *MAP3K21* modulate gene expression^23,39^. An *Alu* variant upstream of *PGR* and a LINE-1 variant upstream of *BDH2* influence their respective gene expression^66^. An *Alu* insertion in an existing enhancer between *DGKE* and *TRIM25* attenuates the enhancer activity of the two genes^23,37^. Conversely, a LINE-1 insertion in an intron of *NEDD4* acts as an enhancer^23^. These examples demonstrate that mobile elements can influence gene expression; some mobile element lineages have the potential to create novel cis-regulatory elements, such as enhancers, and some insertions can attenuate existing cis-regulatory elements. Conceptually, mobile element insertions are unique mutagens compared to single nucleotide mutations because they can add new cis-regulatory elements.

Some studies have evaluated the contribution of SVs to gene expression. Partitioning heritability of gene expression by variant class (i.e., SVs and non-SVs) estimated that SVs account for 6.6% to 8.4% of the variance in total gene expression^19,65^. Since there are fewer SVs than SNVs and indels, the total contribution of SVs, including MEVs, to gene expression is low. However, the insertion of a long sequence may have a greater regulatory impact on the expression of nearby genes than a single nucleotide mutation. A long sequence can encode cis-regulatory elements; mobile elements carry promoter sequences for Pol-III (*Alu*) or Pol-II (LINE-1 and SVA). The insertion of long sequences into existing regulatory elements may disrupt the optimal spacing between regulatory factor binding sites. To gain insight into the strength of the cis-regulatory function of MEVs, we performed a harmonized permutation of variants and found that, in the testis, MEVs are more frequently found in eQTLs than SNVs. This suggests that under specific conditions (e.g., in the testis), MEVs may have greater potential for regulatory functions than SNVs. Nevertheless, as most SNVs in eQTLs are expected to be noneffector variants, most mobile element copies are unlikely to show detectable cis-regulatory effects, i.e., so-called “junk” DNA. Indeed, 92% of the MEVs did not reach statistical significance in our eQTL analysis (this does not necessarily mean that those MEVs are truly junk since they may have gene regulatory effects in tissues we have not investigated and/or may show weak effects that can be captured when a greater number of individuals are used).

We recently performed GWAS for 42 diseases and found that 5 common and relatively common MEVs are associated with diseases^23^. In particular, we found that a LINE-1 insertion in an intron of *NEDD4* is associated with keloids, which are abnormally protruding scars that form during the healing process of a skin injury. The locus carrying the LINE-1 insertion colocalizes with the *NEDD4* eQTL of fibroblasts, and by cellular experiments, LINE-1 was confirmed to function as an enhancer of a short *NEDD4* transcript variant, which is involved in severe keloid formation^67^. This shows that the integration of MEVs in statistical genetics can improve hypotheses about causality and can sometimes pinpoint previously overlooked genetic causes. All 5 disease-related MEVs are noncoding rather than exon-disrupting, although the causality for the remaining 4 MEVs has not been validated experimentally. As exemplified by the LINE-1 insertion in *NEDD4*, it is intriguing to imagine that MEVs are often involved in trait variations driven by the acquisition of new gene regulatory elements, which is known to be true for some lineage-specific enhancers^68^. It is still unclear whether MEVs are more often involved in the emergence of gene regulatory sequences than SNVs; further studies will be required to answer this question.

## Limitations of the statistical genetics of MEVs

One limitation of MEVs in statistical genetics is the sparsity of MEVs. MEVs are 1000 times less prevalent than SNVs. For example, we can only find approximately 2,500 to 3,000 MEVs from one person, while the number of SNVs is approximately 4 to 5 million. Therefore, it is difficult to statistically compare SNVs and MEVs. For example, evaluating the heritability enrichment of MEVs is challenging; thus, the extent to which MEVs contribute to polygenic traits remains an unanswered question.

When comparing variant classes side by side, the difference in genotyping accuracy between variant classes is important. In the case of short-read variant calling, different classes of SVs, such as MEVs, CNVs, and inversions, are often called by different tools specifically developed for each class. In this case, the distributions of genotyping accuracy may be different between variants. While most SNVs are usually accurately called, the genotyping accuracy may be low for non-SNV variants. This may be the case even after quality control, such as the removal of variants violating the Hardy‒Weinberg equilibrium and imputed variants that have a low variance ratio (e.g., Rsq and INFO scores). In such cases, harmonization of variant classes would be needed.

The use of common MEVs does not significantly enhance the discovery of genome loci associated with gene expression and disease. In the GWAS on BBJ, we did not find any LD block that contained only MEVs. In other words, common MEVs were associated with one or more disease-tagged SNV. If the purpose of the analysis is to discover disease-relevant genome loci, the use of MEVs would not be advantageous.

## Future perspectives

Long-read WGS shows promise for discovering and genotyping SVs, including MEVs. Ultimately, long reads may replace short reads; however, short reads are still commonly used, particularly in biobanks, and there are valuable short-read genome resources. Therefore, it is still necessary to develop a reliable protocol to maximize MEV discovery and genotyping accuracy from short-read data. To achieve this goal, first, we need a population-scale ground truth to benchmark population-scale MEV genotyping. This could soon be enabled by a haplotype-resolved long-read WGS in a cohort, such as the 1000 Genomes Project. Because it is unrealistic to expect one tool to confidently identify all MEVs, users may need to think about applying multiple tools and using the union as a confident set^60^.

In addition to common variants, rare variants and somatic mobilizations remain to be evaluated. It is estimated that ~70% of rare diseases are monogenic^69^. The contributions of MEVs to rare diseases have been evaluated in exome sequencing cohorts^35,70^, revealing that investigations of MEVs from exome sequencing can lead to an additional diagnosis for 1 in 3000 to 4000 patients. Considering that MEVs in introns are also involved in aberrant gene expression, the discovery of MEVs from WGS would increase the rate of cases caused by MEVs. Rare insertions can also pose a risk for polygenic diseases. De novo LINE-1 insertions are more often found in genes related to autism spectrum disorder than expected^71^. In addition to heritable mutations, mobilization can occur in somatic cells. LINE-1 transposition can occur in neural progenitor cells^72^ and is widespread in the colorectal epithelium^73^. Aberrant LINE-1 integrations can induce chromosomal rearrangements leading to cancer, and approximately half of all cancers have somatic integrations of mobile elements^53^. Somatic mobilization may be widely involved in aging and disease onset.

In the future, statistical genetics will reveal more associations between MEVs and traits. Similar to the trend of SNVs, it will be important to validate causality by high-throughput assays. Massively parallel reporter assays are a method for evaluating the cis-regulatory effect of variants using a reporter plasmid^74–76^. Currently, they are used for the evaluation of SNVs and small indels. The use of SVs is challenging due to the limited length of the target sequences that can be inserted into the reporter plasmid. *Alu* is relatively short, with most being ~300 bp. Therefore, it may not be unrealistic to evaluate the influence of *Alu* insertions with such a reporter system. However, such a system would be difficult for longer sequences, such as SVA and LINE-1. Another approach is CRISPR-guided genome editing. Middle- to high-throughput CRISPR screens are increasingly applied to evaluate the effects of SNVs^77–79^. Although genome editing was not initially a high-throughput technology, pooled CRISPR perturbation and single-cell analyses have paved the way for more scalable experimental designs^80,81^. Technological advances, such as more efficient large sequence deletions, are necessary to experimentally evaluate the effects of MEVs and SVs.

In addition to loss-of-function mutations and cis-regulatory effects, proteins encoded by mobile elements may have an impact on disease, particularly cancer^82^. Although SVA and *Alu* are noncoding sequences, LINE-1 encodes two proteins, ORF1p and ORF2p. ORF2p exhibits endonuclease activity^83^ and can be a source of DNA damage^84^. Since ORF2p is also encoded in fixed copies, which are far more abundant than polymorphic copies in the human genome, it is still unclear whether the unfixed LINE-1 copies are involved in this mode of action; further research is needed.

Precision medicine is one goal of genetics. MEVs that cause monogenic diseases can be a target of clinical treatment. MEVs in introns occasionally cause aberrant splicing, and such mis-splicing can be restored to normal levels by splicing-switching drugs. For example, antisense oligonucleotides targeting cryptic splicing donor and acceptor sites found in ataxia-telangiectasia patients can restore normal splicing in cell experiments^85,86^, paving the way for the development of genetic therapies targeting MEVs that cause rare diseases. In the future, the identification of MEVs causing monogenic diseases, the development of treatment options for MEVs, and the identification of MEVs amenable to genetic therapy could be the keys to genome medicine for MEVs.
